# Effects of whole seaweed consumption on humans: current evidence from randomized-controlled intervention trials, knowledge gaps, and limitations

**DOI:** 10.3389/fnut.2023.1226168

**Published:** 2023-07-20

**Authors:** João P. Trigo, Marie Palmnäs-Bédard, Mar Vall-Llosera Juanola, Ingrid Undeland

**Affiliations:** Department of Life Sciences, Division of Food and Nutrition Science, Chalmers University of Technology, Gothenburg, Sweden

**Keywords:** macroalgae, seaweed consumption, novel food, risk-of-bias, disease prevention, health promotion

## Abstract

Seaweed is often recognized for its potential health benefits, attributed to its abundance of dietary fibers, protein, and polyphenols. While human observational studies have shown promise, the collective evidence from human intervention trials remains limited. This narrative review aims to comprehensively analyze the effects of seaweed intake on humans, while critically assessing the methodology, including Cochrane risk-of-bias assessment. A search was conducted in online databases, including PubMed, Scopus, and Google Scholar, covering the period from 2000 to May 2023. The focus was on randomized controlled clinical trials (RCTs) evaluating the impact of whole seaweed, either consumed as capsules, integrated into food products or as part of meals. Various health outcomes were examined, including appetite, anthropometric measures, cardiometabolic risk factors, thyroid function, markers of oxidative stress, and blood mineral concentrations. Out of the 25 RCTs reviewed, the findings revealed limited yet encouraging evidence for effects of seaweed on blood glucose metabolism, blood pressure, anthropometric measures, and, to a lesser extent, blood lipids. Notably, these favorable effects were predominantly observed in populations with type-2 diabetes and hypertension. Despite most trials selecting a seaweed dose aligning with estimated consumption levels in Japan, considerable variability was observed in the pretreatment and delivery methods of seaweed across studies. Moreover, most studies exhibited a moderate-to-high risk of bias, posing challenges in drawing definitive conclusions. Overall, this review highlights the necessity for well-designed RCTs with transparent reporting of methods and results. Furthermore, there is a need for RCTs to explore seaweed species cultivated outside of Asia, with a specific emphasis on green and red species. Such studies will provide robust evidence-based support for the growing utilization of seaweed as a dietary component in regions with negligible seaweed consumption, e.g., Europe.

## 1. Background

Seaweed also referred to as macroalgae, has gained attention as a promising food source in Western countries due to its low environmental impact, remarkable nutritional properties, and potentially positive effects on health and disease prevention (1–3). Despite its minimal consumption in most Western countries, seaweed holds a significant dietary role in coastal Asian nations where it is consumed on a daily basis (4). As a result, seaweed has a well-established history of safe consumption and widespread integration into various diets over time.

Seaweed is commonly perceived by consumers as a healthy food and is rich in dietary fibers, such as carrageenan, ulvan and fucoidan found in red, green, and brown species, respectively (5, 6). Furthermore, seaweed is a relatively good source of protein with an average content often ranging from 10 to 30% dry weight (DW) - the upper range being dominated by red and green seaweeds. Also, minor components, such as essential elements (e.g., calcium, iodine, iron), vitamins (e.g., B_12_ and C), polyunsaturated fatty acids (PUFA) (e.g., *n*-3), and phenolic compounds (e.g., phlorotannins) contribute to this positive perception (5). Due to its versatile nutrient composition and distinct flavor properties, whole seaweed is often consumed directly (e.g., in sushi or as a condiment) or, depending on the percentage in the final food formula, as a nutrient or technological food additive. According to literature, seaweed is mainly incorporated in meat- and cereal-based products, and depending on the food product, it can improve the nutrient profile by increasing the proportion of unsaturated fatty acids, provide essential amino acids and increase the dietary fiber content (7–11). Seaweed may also protect the food matrix against oxidation and microbial growth, which could provide additional benefits in terms of extended product shelf-life (12). Optimum incorporation levels have been deemed to range from 3.6–10% for cereal-based products and 1–5% for meat-based products (8–10). Above these values, seaweed often impairs the sensorial quality of fortified products (8–10).

Extensive evidence arising from *in vitro* studies and animal models has suggested that seaweed intake could play a role in biological mechanisms related to health and/or disease prevention. This includes antioxidant-, anticancer-, antidiabetic-, anti-inflammatory-, anti-obesity- and anticoagulant effects caused by numerous bioactive molecules (13). Recent prospective studies have also found associations between seaweed intake and lower mortality as well as decreased risk of developing colorectal cancer, metabolic syndrome, cardiovascular disease and osteoporosis (14–17).

Although several beneficial health effects of seaweed intake have been proposed from observational studies, randomized controlled interventional trials (RTCs), especially those measuring hard endpoints, provide the highest level of evidence among original studies and are ultimately needed to establish causality. To the best of our knowledge, several recent reviews have investigated randomized controlled trials (RCTs) examining the impact of seaweed intake, with a specific emphasis on cardiometabolic diseases and osteoporosis (3, 7, 18–21). The prevailing consensus among these reviews is that the scarcity of literature, coupled with the low scientific quality of current evidence, presents challenges in establishing definitive conclusions. However, there remains a critical need for an in-depth analysis of methodological issues and study design, which, if conducted, can help identify existing knowledge gaps and limitations. Moreover, a substantial proportion of the reviewed RCTs focused on seaweed extracts or isolated components, rather than investigating the effects of whole seaweed. Consequently, a comprehensive understanding of the potential additive or synergistic effects between different components of whole seaweed is currently lacking. Given the identified gaps, there is a need for a review offering a more comprehensive and rigorous analysis of the current state of knowledge regarding the effects of whole seaweed consumption in humans. Therefore, this narrative review aims to address these gaps by evaluating the available evidence regarding the effects of whole seaweed consumption on all measured outcomes thus far. This evaluation was based on the reported findings from RCTs, with a specific focus on summarizing key outcomes and conducting a critical assessment of the methodology and Cochrane risk-of-bias.

## 2. Methods

### 2.1. Eligibility criteria and study selection

**Figure 1:**
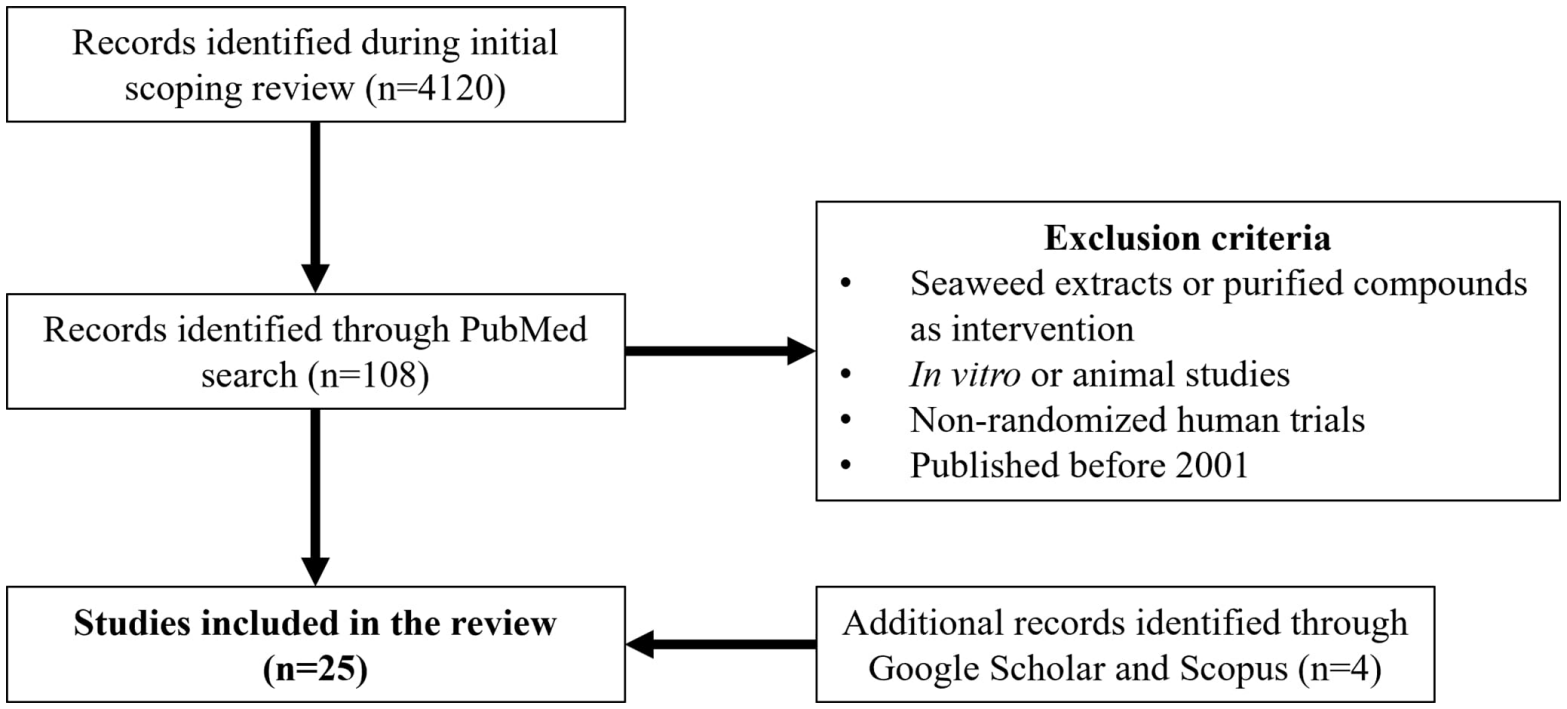
Study selection process of randomized controlled trials (RCTs) examining the effect of seaweed consumption on humans.

A RCT was considered eligible for the present review if it fulfilled the following requirements: (i) any population; (ii) whole seaweed or fermented products thereof as intervention (including products made with whole seaweeds, e.g., bread); (iii) outcomes including blood glucose response (e.g., fasting levels, postprandial glucose/insulin), blood lipids, blood pressure, thyroid function, urinary iodine, anthropometric measurements, markers of oxidative stress, subjective appetite; (iv) parallel or cross-over study design; (v) a control group included; (vi) written in English; (vii) published from 2000 (inclusive) to June 2022; the year 2000 was defined as the lower cut-off since previous reviews did not find any RCT that tested the effect of whole or fermented seaweed intake before that year (3, 7, 18–21). After an initial literature review that resulted in 4120 records, further searches, including specifying outcomes based on this *a priori* knowledge, were performed in June 2022 using the CADTH search filter for randomized controlled trials indexed in PubMed (22) and a search string for seaweed (“seaweed OR macroalgae OR algae”). Also, multiple combinations were conducted between the previous seaweed string and terms related to RCTs and outcomes such as “health,” “cross-over,” “parallel,” “blind*,” “intervention,” “randomize*,” “control*,” “clinical,” “human*,” “placebo,” “trial,” “diabetes,” “appetite,” “blood,” “blood pressure[MeSH],” “lipid[MeSH],” “blood glucose [MeSH],” “insulin,” “minerals[MeSH],” “thyroid[MeSH],” “iodine[MeSH], “obesity[MeSH],” “antioxidant[MeSH].” Overall, after removing duplicates, these searches resulted in 108 articles, whereof 87 were excluded due to the following reasons: (i) seaweed extracts or purified compounds as intervention; (ii) *in vitro* and/or animal studies; (iii) non-randomized clinical trials, which included two trials (23, 24) and the supplementation study of Combet et al. (25). Further searches using Google Scholar and Scopus identified an additional 4 articles (26–29), bringing the total number of RCTs included in this review to 25. None of the eligible RCTs studied the effect of seaweed intake on disease risk, e.g., disease risk scores or indices. Additionally, a recent search was performed in PubMed, covering the period from 2022 to May 2023, yielding 215 records. However, none of these records met the predefined inclusion criteria. Figure 1 summarizes the whole process of study selection.

### 2.2. Risk-of-bias assessment

**Figure 2:**
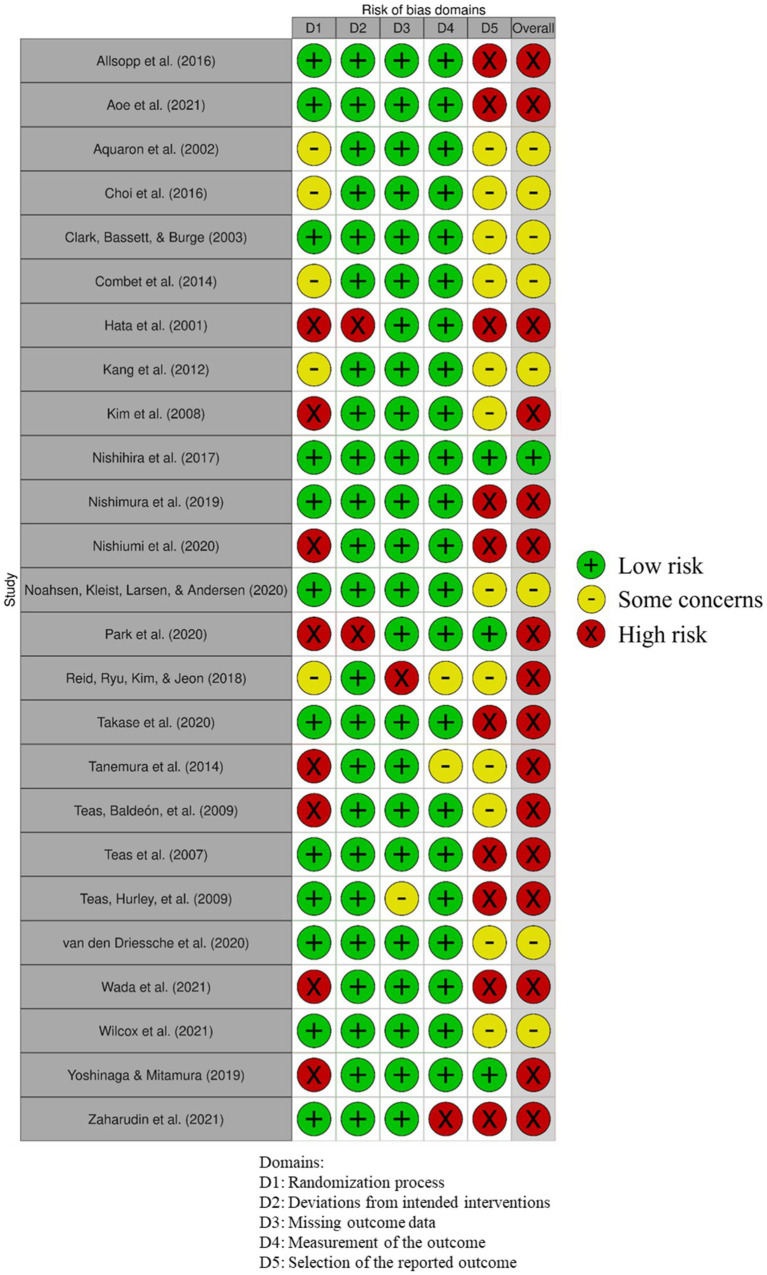
Cochrane individual risk-of-bias judgment of all identified randomized controlled trials (RCTs).

The risk-of-bias of the compiled studies was conducted according to the revised Cochrane risk-of-bias tool for randomized trials (RoB 2) (30). This tool considers 6 risk-of-bias domains: randomization process; deviations from the intended interventions; missing outcome data; measurement of the outcome data; selection of the reported result; and overall bias. According to the tool, each domain was categorized as “low risk,” “some concerns,” and “high risk.” The bias assessment was first independently conducted (in a blinded manner) by the first and second authors (JT and MP-B). Upon finalization, the individual assessments were compared, and conflicting assessments were resolved upon discussion, reaching a unanimous decision. Figure 2 was generated using robvis tool developed by McGuinness and Higgins (31).

## 3. Impact of seaweed consumption on humans

### 3.1. Overview of the identified RCTs

From the initial 4,120 literature records, 25 RCTs fulfilled our criteria, including 11 cross-over studies and 14 with parallel study design. The most studied primary outcomes were blood glucose metabolism and blood lipids, each represented by four RCTs, followed by urinary iodine (*N* = 3), blood pressure, thyroid function, oxidative stress (*N* = 2, each), and anthropometry, blood minerals concentration, and biomarkers of immunology and inflammation (*N* = 1, each). Moreover, for secondary and exploratory outcomes we found the following number of articles per category: blood lipids (*N* = 8), thyroid hormones (*N* = 7), blood glucose metabolism, blood pressure (*N* = 6, each), anthropometric measures (*N* = 5), urinary iodine (*N* = 3), antioxidant effects, as well as appetite scores and neurological/physical performance (*N* = 2, each), blood minerals levels, biomarkers of inflammation, and urinary proteins (*N* = 1, each). Only 8 of the identified RCTs were registered in databases such as clinicaltrials.gov, umin.ac.jp/ctr/, isrctn.com, and cris.nih.go.kr (website not available as of October 2022), whereof 5 were registered *a priori* and locked, unaltered or without changes that may impact on bias assessment (26, 28, 32–34).

As listed in Table 1, half of the RCTs were conducted either in Japan (*N* = 9) or South Korea (*N* = 5), where consumption of seaweed is widespread. The remaining RCTs were mainly conducted in Western countries, which included the United States (*N* = 3) and United Kingdom (*N* = 3) as well as Greenland, Belgium, Canada, Denmark, and the Netherlands (*N* = 1 per country).

**Table 1:** Summary of the identified randomized controlled trials (RCTs) investigating the influence of seaweed intake on specific health outcomes.

Primary outcome	Reference	Country	Type of randomized study	Study population	Randomized subjects (age range)	Seaweed intervention	Control	Seaweed quantity and frequency	Compliance measured	Specific primary outcome	Significant effects: seaweed vs. control	Significant effects in seaweed arm: baseline vs. final	Overall risk-of-bias
Anthropometry	Aoe et al. (35)	Japan	Parallel-group, double-blind	With overweigh	23 men and 25 women (20–59)	Capsules containing boiled, dried *Laminaria japonica*	Similar capsules containing more maltitol and microcrystalline cellulose	6 g DW/day for 8 weeks	*Not mentioned*	*Not specified*	↓Body fat percentage (only in men, *p* < 0.05)	↓LDL-C (*p* < 0.05)	High
Blood glucose metabolism	Wilcox et al. (39)	UK	Acute study, cross-over, double-blind	Healthy	7 men and 3 women (19–29)	Bread enriched with dried *Ascophyllum nodosum* or *Fucus vesiculosus*	Similar bread without seaweed	0.5 and 2 g DW for each species/meal	Conducted in feeding facilities	Postprandial blood glucose	No significant changes reported.	*Not applicable*	Some concerns
	Yoshinaga and Mitamura (32)	Japan	Acute study, cross-over, open-label	With untreated type 2 diabetes	15 men and 11 women (20–59)	Rice with dried *Undaria pinnatifida*	Rice without seaweed	4 g DW	Conducted in feeding facilities	Postprandial blood glucose and insulin, glucose AUC, insulin AUC	↓Postprandial blood glucose and insulin after 30 min (*p* < 0.01; *p* < 0.05) ↓Blood glucose AUC	*Not applicable*	High
	Zaharudin et al. (38)	Denmark	Acute study, cross-over, 3-way blinded	Healthy	9 men and 11 women (20–50)	Meal containing corn starch and re-hydrated dried *Laminaria digitata* or *Undaria pinnatifida*	Similar meal with pea protein replacing seaweed	5 g DW/meal	Conducted in feeding facilities	Postprandial blood glucose	↓Postprandial blood glucose between 40 and 90 min (in participants with ≤63 kg, *p* < 0.05) ↓Glucagon-like peptide-1 response after intake of *L. digitata* (*p* < 0.05) ↑Satiety and fullness iAUC (*p* < 0.01)	*Not applicable*	High
	Tanemura et al. (41)	Japan	Acute study, cross-over, open-label	Healthy	8 men and 4 women (av. 25 years old)	Breakfast served with raw *Undaria pinnatifida*	Similar breakfast without seaweed	70 g FW/ meal	Conducted in feeding facilities	Postprandial blood glucose and insulin	No significant changes	*Not applicable*	High
Blood lipids	Nishimura et al. (26)	Japan	Parallel-group, double-blind	Healthy	33 men and 33 women (30–70)	Capsules containing boiled dried *Saccharina japonica*	Capsules containing dextrin instead of seaweed	2 g DW/day for 6 weeks	*Not mentioned*	Total cholesterol, LDL-C, HDL-C, triglycerides, LDL- C/HDL-C ratio, and non-HDL	↓Adiponectin (p < 0.05)	No significant changes reported	High
	Takase et al. (33)	Japan	Parallel-group, double-blind	With hyper-cholesterolemia	34 men and 70 women (20–59)	Capsules dried containing *P. palmata*	Placebo capsules	2 g DW/day for 8 weeks	*Not mentioned*	LDL-C	↑Urine iodine/Cr ratio, TSH (*p* < 0.01) ↓Triglycerides, triglyceride/HDL-C ratio (only in women, *p* < 0.05)	↓Body weight, BMI, urine iodine (*p* < 0.05) ↓Glycated albumin (*p* < 0.01) ↑LDL, non-HDL (*p* < 0.05) ↑Urine iodine/Cr ratio, TSH (*p* < 0.01)	High
	Van den Driessche et al. (34)	the Netherlands	Cross-over, double-blind	Healthy	15 men and 20 women (18–70)	Capsules containing dried *Undaria pinnatifida*	Placebo capsules; seaweed replacer not mentioned	4.8 g DW/day for 2.4 weeks	Participants were asked to return empty sachets and unused capsules	Serum cholesterol-standardized campesterol	No significant changes reported	No significant changes reported	Some concerns
	Nishiumi et al. (46)	Japan	Cross-over *(no mention regarding type of blinding)*	Healthy	20 men and 28 women (35, 39–85)	Roasted *Laminaria japonica*	*Not applicable*	6 g DW/day for 4 weeks	*Not mentioned*	*Not specified*	*Not applicable*	No significant changes reported	High
Blood minerals	Park et al. (69)	South Korea	Parallel-group, open-label	With low calcium intake	29 women (18–34, 36–40)	Days 0–5: dried *Undaria pinnatifida* and *Porphyria* (ratio 9:1) included in basal diet Days 6–19: noodles containing 98% dried *U. pinnatifida* included in basal diet	Habitual diet	Days 0–5: 4 g DW/day Days 6–19: 2 packs of noodles/ every 3 days	Days 0–5: conducted in feeding facilities Days 6–19: self-report and number of packs returned	*Not specified*	Days 0–5: no significant changes Days 6–19: no significant changes	Days 0–5 (*p* < 0.05): ↑Serum 1,25-dihydroxyvitamin D ↓Serum parathyroid hormone Days 6–19 (*p* < 0.05): ↑Serum calcium	High
Blood pressure	Hata et al. (27)	Japan	Parallel-group *(no mention regarding type of blinding)*	With hypertension	12 men and 24 women (35, 39, 41–85)	Capsules containing dried *Undaria pinnatifida*	No treatment	5 g DW/day for 8 weeks	Interviewing the participants at each hospital visit	*Not specified*	↓Diastolic blood pressure (*p* < 0.05)	↑Free fatty acids (*p* < 0.05) ↓Uric acid, platelets, systolic and diastolic blood pressures (*p* < 0.05)	High
	Wada et al. (36)	Japan	Parallel-group, open-label	Healthy	39 boys and 42 girls (4, 5)	Lunch served or not with roasted red nori in sheets	Similar lunch without seaweed	1.76 g DW /day for 10 weeks	*Not mentioned*	*Not specified*	↓Diastolic blood pressure (only in boys, p < 0.05)	No significant changes reported	High
Oxidative stress	Kang et al. (71)	South Korea	Parallel-group, double-blind	With high level of serum gamma-GT	48 men (25–60)	Capsules containing fermented dried *Laminaria japonica*	Similar capsules containing more lactose	1.5 g DW/day for 4 weeks	*Not mentioned*	*Not specified*	↓Serum gamma-glutamyl transferase, MDA (p < 0.05) ↑Superoxide dismutase, catalase (p < 0.05)	↓Serum gamma-glutamyl, MDA (p < 0.05) ↑Superoxide dismutase, catalase, glutathione peroxidase (p < 0.05)	Some concerns
Thyroid function & urinary iodine	Clark et al. (82)	USA	Parallel-group, double-blind	Healthy	18 men and 18 women (21–55)	Capsules containing dried kelp	Similar capsules containing dried alfalfa as seaweed replacer	Low dose: 1.2 g DW/day for 4 weeks High dose: 2.4 g DW/day for 4 week	*Not mentioned*	TSH, thyrotropin-releasing hormone, T3, FT_4_	*Not analyzed*	↑Urinary iodine (p < 0.01) ↑TSH (p < 0.05) ↑Thyrotropin-releasing hormone (only high dose, *p* < 0.001) ↓Total T_3_ (only high dose, p < 0.05)	Some concerns
	Noahsen et al. (40)	Greenland	Acute study, parallel-group, single-blinded	Healthy	2 men and 4 women (23–32)	Sushi meal with salad containing blanched fresh *Fucus vesiculosus*	Same sushi meal without salad	25 g FW/meal	*Not mentioned*	Urinary iodine excretion, TSH, fT_4_,	↑Urinary iodine (p < 0.001)	↑Urinary iodine (p < 0.001) ↑TSH (p < 0.05)	Some concerns
		Greenland	Acute study, parallel-group, single-blinded	Healthy	3 men and 3 women (24–65)	Sushi meal with salad containing fresh Japanese seaweed	Same sushi meal without salad	25 g FW/meal	*Not mentioned*	Urinary iodine excretion, TSH, fT_4_,	*Not mentioned*	↑Urinary iodine (p < 0.001) ↑TSH (p < 0.05)	
Estrogen & phytoestrogen	Teas et al. (51)	USA	Cross-over, double-blind	Healthy, postmenopausal	15 women (av. 59 years old)	Capsules containing dried *Alaria esculenta*	Capsules containing maltodextrin as seaweed replacer	5 g DW/day for 6 weeks	Daily journal and measurement of urinary iodine	*Not specified*	↓Serum estradiol as function of the seaweed intake per body weight (p < 0.01)	*Not mentioned*	High
Biomarkers	Nishihira et al. (28)	Japan	Parallel-group, double-blind	Healthy	10 men and 48 women (35–65)	Capsules containing dried *Kjellmaniella crassifolia*	Capsules containing dextrin as seaweed replacer	0.8 g DW/day for 8 weeks	*Not mentioned*	Natural killer cell activity	↓Natural killer cell activity (only in subjects with elevated baseline NK cell activity, p < 0.05)	No significant changes	Low
	Allsopp et al. (43)	Northern Ireland (UK)	Parallel-group, double-blind	Healthy	17 men and 18 women (18–65)	Bread enriched with dried *Palmaria palmata*	Similar bread without seaweed	5 g DW/day for 4 weeks	Participants were asked to return unused bread	Serum C-reactive protein	↑Serum C-reactive protein (p < 0.05) ↑Triglycerides (p < 0.05) ↑TSH (p < 0.05)	↑Serum C-reactive protein (p < 0.05) ↑Triglycerides (p < 0.05) ↑TSH (p < 0.05)	High
Urinary iodine	Aquaron et al. (42)	Belgium	Acute study, cross-over, double-blind	With mild iodine deficiency	9 men (age not mentioned)	Capsules containing dried *Laminaria hyperborea* or *Gracilaria verrucosa*	Capsules containing lactose as seaweed replacer	0.38 g DW for each species for 1 day	*Not mentioned*	Urinary iodine	↑Urinary iodine	*Not applicable*	Some concerns
	Combet et al. (25)	UK	Acute study, cross-over *(type of masking not mentioned)*	Healthy	22 women (22–34)	Capsules containing dried *Ascophyllum nodosum*	Capsules containing potassium iodide	1 g DW/day for 1 day	*Not mentioned*	Urinary iodine	↓Total urinary iodine excreted over 24 h (p < 0.001)	↑Total urinary iodine excreted (p < 0.05)	Some concerns
Not specified	Teas et al. (57)	Ecuador	Cross-over, double-blind	At least one condition of metabolic syndrome	13 men and 14 women (av. 46 years old)	Capsules containing dried *Undaria pinnatifida*	Group 1: capsules containing maltodextrin as seaweed replacer Group 2: no treatment	Group 1: placebo for 4 weeks, followed by 4 g DW/day for 4 weeks Group 2: 4 g DW/day for 4 weeks, followed by 6 g DW/day for 4 weeks	*Not mentioned*	*Not specified*	Group 1: no significant changes reported versus placebo Group 2: *not applicable*	Group 1: ↓Waist circumference (p < 0.01) Group 2: ↓Waist circumference (only in women after high dose; p < 0.01) ↓Systolic blood pressure (after high dose, p < 0.01)	High
	Kim et al. (63)	South Korea	Parallel-group, open-label	With type 2 diabetes	9 men and 11 women (35, 41–70)	Capsules containing equal parts of dried *Saccharina japonica* and dried *Undaria pinnatifida*	*Details not mentioned*	1.7 g DW/day for 4 weeks	*Not mentioned*	*Not specified*	↓Postprandial blood glucose (p < 0.05) ↓Triglycerides, LDL-C (p < 0.05) ↓TBARS (p < 0.05) ↑Catalase, glutathione peroxidase (p < 0.05)	↓Fasting blood glucose (p < 0.01) ↓Postprandial blood glucose and triglycerides (p < 0.05) ↑HDL-C (p < 0.05)	Some concerns
	Teas et al. (53)	USA	Cross-over, double-blind,	Healthy, postmenopausal	25 women (av. 58 years old)	Capsules containing dried *Alaria esculenta*	Capsules containing maltodextrin as seaweed replacer	5 g DW/day for 6 weeks	*Not mentioned*	*Not specified*	↑Urinary iodine, TSH (p < 0.01)	No significant changes reported	High
	Reid et al. (37)	South Korea	Parallel-group, double-blind	Healthy	60 participants (av. 74 years old)	Capsules containing fermented dried *Saccharina japonica*	Capsules containing sucrose as seaweed replacer	1.5 g DW/day for 6 weeks	*Not mentioned*	*Not specified*	↑Raven’s test, iconic memory tests, serum GSR, serum SOD, serum IGF-1 (p < 0.01) ↑Numerical memory test, serum GPx, serum BDNF (p < 0.05) ↓6-min walk, serum TBARS, serum 8-oxoDG (p < 0.01)	*Not mentioned*	High
	Choi et al. (29)	South Korea	Parallel-group, double-blind	Healthy	21 women (53–63)	Capsules containing fermented dried *Saccharina japonica*	Similar capsules containing more lactose or sucrose capsules; unclear which capsule was used	1.0 g DW/day for 8 weeks	*Not mentioned*	*Not specified*	↑Human growth hormone, brain derived neurotrophic factor (p < 0.001) ↑Total lean mass (p < 0.01) ↓Angiotensin converting enzyme (p < 0.01)	↓Angiotensin converting enzyme (p < 0.01) ↓Total triglycerides, total fat (p < 0.05) ↑Human growth hormone, insulin-like growth hormone-1, lower limb strength measured as peak torque normalized to BW at 60 ° s^−1^ flexion (p < 0.05)	Some concerns

Most RCTs investigated effects from interventions in healthy subjects. Among these RCTs, one was conducted with healthy postmenopausal women, and three of them were in subjects who had low calcium or iodine intake, or high levels of serum gamma-glutamyl transferase. In the remaining studies, subjects presented with type 2 diabetes or risk factors for cardiometabolic diseases, including overweight, hypercholesterolemia, and/or hypertension. On average, the study population of each RCT was composed of 58 ± 27% females (range: 0–100%) and the most predominant age range was 18 to 70 years old; exceptions to this include RCTs conducted exclusively in children (36) and elderly people (37). Excluding the *N* = 7 acute studies focusing on postprandial effects (25, 32, 38–42), the average sample size for parallel and cross-over studies was 51 (range: 20–104) and 30 subjects (range: 15–48), respectively. Only 9 RCTs reported a power calculation (25, 26, 28, 33, 34, 36, 40, 43), thus it is unknown if the sample size was properly dimensioned to detect statistically significant differences for their respective outcomes. Lastly, the average study length was 6.4 (range: 2.7–10) and 4.5 weeks (range: 2.4–6) for parallel and cross-over studies, respectively.

#### 3.1.1. Analysis of seaweed treatment

Most studies investigated brown seaweed species; only three studies used red seaweeds (33, 36, 43) and none investigated green seaweeds. In RCTs conducted in Asia, species *Saccharina japonica* and *Undaria pinnatifida*–commonly known as Kombu and Wakame, respectively–predominated and are traditionally eaten in this region. In contrast, RCTs performed in European countries mainly tested *Ascophyllum nodusum* or *Fucus vesiculosus*, while *U. pinnatifida* was used in most RCTs performed in the United States. It is also worth noting we did not find any RCTs investigating the effects of whole *Saccharina latissima* (sugar kelp), which is one of the most cultivated seaweed species in Europe (44, 45).

The average daily dosage used in RCTs comprising dried and wet seaweed was 2.9 g (range: 0.4–6.0) and 26.3 g (range: 5.0–70), respectively. According to a recent survey conducted on the Japanese population (*N* = 240; 50% female; BMI 23 ± 2.9 kg/m^2^) the average intake of seaweed was 1.7 ± 1.4 g dried plus 8.5 ± 8.4 g wet (4). One can sum both quantities by assuming a proximate moisture content of 90%, thus resulting in an average daily intake of 2.6 g DW or 25.5 g FW. These values are comparable to the average daily dosage selected in most RCTs (Table 1). This is important as similar doses facilitate the interpretation of results and imitate real-life scenarios. Notwithstanding, several RCTs in Table 1 hypothesized that seaweed would exert hypolipidemic effects due to its richness in dietary fiber and n-3 PUFA (33, 34, 43, 46). However, only pharmacological doses of 2–3 g/day of these fatty acids have been proven to reduce triglycerides levels (47). Therefore, massive amounts of seaweed would be needed to deliver those doses - total lipids rarely exceed 4.5% DW in seaweed (5), which can be compared with 1.5–30% lipids per wet weight (ww) in fatty fish (48, 49).

Regarding mode of delivery, most RCTs (*N* = 16) opted for encapsulated seaweed, followed by including it in meals (*N* = 7) e.g., as salad and incorporation in either bread (*N* = 2) or noodles (*N* = 1). While encapsulation allows for blinding in theory (Table 1), blinding can in our opinion easily become compromised in RCTs investigating effects of seaweed. This is because seaweed contains a complex mixture of volatile compounds (50) that often result in a unique and strong flavor making seaweed capsules easily distinguishable from placebo. As far as we know, only one study (35) using encapsulated seaweed sought to overcome this issue by incorporating 0.1% (w/w) of “kelp flavor powder” in the placebo capsules. The remaining RCTs did not report on flavor aspects or comparisons between treatment and placebo, but some RCTs mentioned similarities in color (33) and appearance (26, 51). This is also important since color and visual appearance can lead to failure to blind or unwanted unblinding of investigators, study personnel and/or participants. Another important aspect related to the mode of delivery, concerns to the nutrient/ingredient composition of the placebo capsules. On this matter, alfalfa, dextrin, maltitol, maltodextrin, microcrystalline cellulose, lactose, soy protein, and sucrose were selected as seaweed replacers, while some studies compared meals with or without seaweed (Table 1). Depending on the research question, leafy vegetables such as head lettuce or white cabbage can also be good candidates for a control diet due to similar chemical composition, such as dietary fiber content since it is close to the one found in, e.g., *L. digitata and U. pinnatifida*–around 30% DW (5, 52).

A total of 22 RCTs (Table 1) used dried seaweed, whereas the remaining trials chose re-hydrated (40, 41, 43) or fresh (38). Within each alternative (i.e., dried and wet/rehydrated), we found large variations in how the seaweed was pre-treated or dried, which ultimately can alter the nutritional composition and potential health effects. Specific pre-treatments were boiling (35), desalting (27), washing (26, 33), and/or salting (26) and drying techniques included, e.g., roasting (36, 46) or sun-drying (53) - interestingly, it was unclear if roasting was performed as a drying technique *per se* or as a treatment following, e.g., sun-drying. Based on these large variations, care should be taken when comparing findings from RCTs testing the same species since the retention of, e.g., total monosaccharides, essential amino acids, PUFA and essential elements can change as a function of the pre-treatment and drying technique (54–56). Unfortunately, none of the reviewed RCTs disclosed the complete nutritional composition of the tested seaweeds (Table 1). Another aspect limiting direct comparisons across RTCs that investigated the same species concerns which anatomical part of the seaweed was used. For instance, one study (57) only used sporophylls from *U. pinnatifida* since this anatomical part was ascribed as having higher levels of fucoidan (58), while for the same primary outcome, another study (27) tested *U. pinnatifida* as a whole.

### 3.2. Effects on blood lipids

Blood lipid abnormalities such as hypertriglyceridemia and hypercholesterolemia are risk factors for several cardiometabolic diseases, including cardiovascular diseases, the leading cause of death worldwide–accounting for nearly 19 million deaths in 2019 (59, 60). Diet is one of the key modulators of blood lipids, and increased consumption of dietary fibers and an altered consumption of dietary fats can have an impact on blood lipids (61, 62).

In this review, 12 RCTs measured blood lipids, whereof 4 studies had blood lipids as the primary outcome (26, 33, 34, 46). No study reported effects at post-intervention between intervention and control groups. Compared to the baseline, three RCTs reported significant changes in blood lipids following the consumption of seaweeds (33, 43, 63), more specifically, two studies found a reduction in triglycerides and/or cholesterol and one found elevations in triglycerides. It should be noted that comparisons against baseline can be biased and misleading (64).

In brief, serum triglycerides significantly decreased after consumption of 2 g DW of *P. palmata* per day for 8 weeks in individuals with hypercholesteremia, however only in women (33). Compared to baseline, triglycerides levels decreased by 9.0 mg/dL (range: −25.0, 5.0) for the seaweed group and 1.0 mg/dL (range: −11.0, 19.0) for the placebo group consuming capsules only containing porcine gelatin. Similarly, compared to the baseline, one study (63) observed a significant decrease in serum triglycerides (−59.4 vs. −4.3 mg/dL in the control) and serum LDL (−31.1 vs. +1.7 mg/dL). Moreover, in the seaweed intervention group, HDL-cholesterol levels increased from 37.1 ± 3.2 mg/dL at baseline to 44.6 ± 2.9 mg/dL post-intervention. In this trial, participants with type 2 diabetes were provided 1.7 g DW seaweed/day for 4 weeks through capsules containing *S. japonica* and *U. pinnatifida* in a 1:1 ratio (63). Conversely, another study (43) observed a 30 mg/dL increase in serum blood triglycerides in healthy subjects after 4-week consumption of bread containing 5 g FW per day of *P. palmata*, compared to the baseline. Differences in post-intervention triglyceride levels were limited (122.1 ± 38.1 for the seaweed group and 117.7 ± 84.1 mg/dL for the control groups) and only statistically significant after controlling for age, sex, BMI, and smoking status. No significant changes were reported for total cholesterol compared to the control group.

To sum up, there is limited and contradictory evidence for effects on blood lipids. Differences were mainly found when comparing changes to baseline, and were in one case, also gender dependent. Thus, these findings should be interpreted with caution (64) and provide overall weak evidence for favorable effects on blood lipids.

### 3.3. Effects on blood glucose metabolism

Hyperglycemia, insulin resistance and prediabetes are reversible conditions of aberrant glucose regulation that if left untreated can lead to chronic type-2 diabetes and diabetic complications. The global prevalence of pre-diabetes and type 2 diabetes is high, with around 791 million people in 2019 (65). Diet can mitigate hyperglycemia and attenuate blood glucose response (66).

Ten RCTs analyzed blood glucose metabolism, whereof 4 reported statistically significant differences compared to the control diet post-intervention (32, 38, 41, 63). In the study by Yoshinaga and Mitamura (32), subjects with untreated type 2 diabetes had lower glucose and insulin levels after 30 min of ingesting rice and 4 g DW of *U. pinnatifida*, but not at the other time points, compared to the control meal without seaweed. These findings should be interpreted with caution as it is unclear if the statistical differences were first discovered based on significant interaction term between time × treatment followed by *post hoc* comparisons to reveal which time points differed. Another study (63) reported–after a suitably paired *t*-test–a significantly lower postprandial glucose after 120 min post seaweed consumption in subjects with type 2 diabetes (203.1 ± 12.3 vs. 254.4 ± 22.8 mg/dL in the control). The supplementation was 1.7 g DW/day for 4 weeks of capsules containing equal parts of *Saccharina japonica* and *U. pinnatifida* compared to placebo capsules of unknown composition (63). Reduced blood glucose levels were also found in healthy participants 30 min post-consumption of a relatively high dose of *U. pinnatifida* (70 g FW) as part of a meal containing rice, soybeans, potatoes, and broccoli, in comparison to the same meal without seaweed (41). Lastly, a daily dose of 5 g DW of either *Laminaria digitata* or *U. pinnatifida* served with corn starch did not change postprandial glucose levels when compared to corn starch served with pea protein in healthy normoglycemic individuals (38). In contrast, glucagon-like peptide-1 was higher in the intervention group and C-peptide and insulin were only significantly lower compared to control after controlling for type of meal, intervention time, and body weight (38). An exploratory sub-group analysis indicated differential effects on glucose in a subgroup with lower body weight and predominately women (38).

Overall, there is indicative evidence to suggest that seaweed may reduce blood glucose response directly following consumption, especially in individuals with type-2 diabetes. However, findings were mainly reported for single time points and most studies did not find any effects on fasting levels, insulin, and measures following longer-term consumption.

### 3.4. Effects on blood pressure

Elevated blood pressure, i.e., hypertension and pre-hypertension is a key risk factor for cardiovascular disease, e.g., stroke and ischaemic heart disease. A recent report estimated that the global prevalence of hypertension in adults aged 30–79 years was around a third (67). Seaweed has been suggested to have blood pressure-lowering effects due to polysaccharides such as alginate that bind to sodium in the gastrointestinal tract, thereby lowering sodium absorption through its excretion (27, 57). Other potential mechanisms may include specific phlorotannins (exclusive phenolic compounds found in seaweed) that act as inhibitors of the angiotensin-converting enzyme, which indirectly modulates blood pressure levels (68).

Out of the 8 RCTs measuring blood pressure (Table 1), only two that had blood pressure as primary outcome reported significantly lower systolic and/or diastolic blood pressure compared to the control (27, 36). However, these results were only observed in certain sub-groups, at specific time points and/or after adjusting for baseline blood pressure.

In a parallel and case-controlled study, one study (27) tested the effect of 5 g DW/day of *U. pinnatifida* for 8 weeks in subjects with hypertension. No differences were observed after 8 weeks compared to the control group. Instead, systolic blood pressure was significantly lower in the seaweed group after 4 weeks compared to the control group. Compared to baseline, only the seaweed group had lower systolic and diastolic blood pressure after 4 weeks and post-intervention (27). In a cross-over study (57), adult participants with at least one symptom of metabolic syndrome were subjected to 4 g DW/day of *U. pinnatifida* for 4 weeks, followed immediately by 6 g DW/day for an additional 4 weeks. Compared to baseline, systolic blood pressure decreased, but only when stratifying based on baseline systolic blood pressure (57). Lastly, a study (36) observed a reduction in diastolic blood pressure in 5-year-old healthy boys after 10 weeks of consuming 1.76 g seaweed DW/day, but only after adjusting for baseline blood pressure.

It is worth noting that the last two cited studies (27, 57) investigated relatively high doses of 4–6 g DW/day in participants with hypertension or metabolic syndrome, whereas most null results were found in studies with doses lower than 5 g DW/day and participants with normal blood pressure (26, 28, 33, 34) (Table 1).

In summary, there is suggestive evidence that seaweed may decrease blood pressure among participants with high blood pressure when consumed at doses higher than 4 g DW/day for at least 4 weeks.

### 3.5. Effects on blood minerals concentration

Two RCTs (Table 1) analyzed blood minerals as primary and secondary/exploratory outcomes, respectively (27, 69). One RCT examined calcium, a macro-mineral crucial for bone health and found in relatively high amounts in brown seaweed (70). In this study, the authors investigated whether *U. pinnatifida* incorporation in the habitual diet would lead to an increase in serum calcium levels in women with low calcium intake (69). Participants consumed 4 g DW seaweed/day equivalent to 133 mg of calcium for 5 days, followed by 2 packs of seaweed noodles every third day for 19 days, providing around 35 mg of calcium per day; but an undisclosed amount of seaweed. No changes were found in serum calcium or three serum biomarkers for calcium intake (25-hydroxy vitamin D3; 1,25-dihydroxyvitamin D; parathyroid hormone) compared to the habitual diet (69). The other study (27) reported similar findings for sodium, potassium, and chloride in subjects with hypertension that consumed 5 g/day of *U. pinnatifida* for 8 weeks versus a parallel group with no intervention.

Based on these two studies, there is no support that *U. pinnatifida* can be used to increase blood levels of calcium, sodium, potassium, or chloride.

### 3.6. Effects on markers of oxidative stress

Seaweed, especially brown species, has been hypothesized to counteract oxidative stress–defined as an imbalance between the generation of free radicals and their scavenging or termination by enzymatic and non-enzymatic antioxidants–partially due to their high content of phenolic compounds and sulfated polysaccharides (37, 63, 71). Oxidative stress has been related to the pathogenesis of several age-related diseases, such as Alzheimer’s disease, cardiovascular diseases, cancer, and rheumatoid arthritis (72), albeit with controversy (73).

Four RCTs (Table 1) examined markers of oxidative stress, such as oxidation products and antioxidant enzyme activity, and three showed favorable effects from the seaweed intervention compared to the control (37, 63, 71). In one study (71), individuals with elevated serum gamma-glutamyl transferase–a general marker of liver damage - were provided 1.5 g DW/day of fermented *S. japonica* to investigate whether seaweed could improve liver status through antioxidant effects. After a 4-week intervention, gamma-glutamyl transferase levels were significantly lower than in the placebo group, 78.7 vs. 116.8 U/L, respectively. This was accompanied by a significant increase in the activity of endogenous antioxidant enzymes such as serum superoxide dismutase (SOD) and catalase (CAT), while glutathione peroxidase (GPx) activity remained unaffected (71). Similarly, a longer intervention (6 weeks) with 1.5 g DW/day of fermented *S. japonica* with elderly subjects resulted in significant increases in GPx, SOD, and glutathione reductase activities as well as a reduction in blood markers related to lipid oxidation (measured through thiobarbituric acid reactive substances - TBARS) and DNA oxidation (8-oxoDG), when compared to the control group provided with sucrose capsules (37). Intake of encapsulated *S. japonica* blended with *U. pinnatifida*, providing 1.7 g DW seaweed/day, resulted in similar trends for CAT and GPx activities as well as TBARS in subjects with type-2 diabetes (63). Conversely, in healthy individuals, a higher dose of red seaweed (*P. palmata*, 5 g DW/day) did not impact the ferric-reducing ability of plasma (43).

In summary, intake of brown seaweed, namely *S. japonica* with or without *U. pinnatifida,* increased activity of several antioxidant enzymes and/or decreased levels of markers of DNA and lipid oxidation. No effect was seen after consumption of the red seaweed *P. palmata*, which contains a lower concentration of polyphenols compared to brown species (5).

### 3.7. Effects on anthropometric measurements

Modulating diet is one of the most effective strategies to prevent overweight and obesity, both major risk factors for diseases such as cardiovascular diseases and type-2 diabetes as well as premature death (74).

One out of 6 RCTs measuring anthropometric measurements (Table 1) reported significantly lower measures after seaweed consumption, compared to the control (35). In one study (35), body fat percentage was significantly lower for men (but not women) with overweight after 8 weeks of consuming 6 g DW/day of *L. japonica*, when compared to the placebo group (calorie content of placebo not provided). Trends of lower BMI and body weight were non-significant (35). Another study (57) reported a significant reduction in waist circumference after *U. pinnatifida* intake (4 g DW/day), but only versus baseline, and there were no changes in BMI or body weight. Importantly, none of the 6 studies aimed at having weight-stable participants through the intervention.

At present, there is weak evidence to suggest that seaweed intake may lead to reductions in anthropometric measures. Studies reporting on such findings intervened with a relatively high dose (≥ 4 g DW/day) of seaweed and only found changes in specific measures and one case was gender dependent.

### 3.8. Effects on subjective appetite

Appetite is central to habitual eating, playing a crucial role in the selection of foods and amounts consumed. Thus, effects on appetite may influence subsequent body weight and composition and weight-related disorders (75, 76). Two single-meal studies (Table 1) tested if seaweed, consumed as part of meals, influenced appetite measured by visual analog scales (38, 41).

One study (38) investigated effects of two brown seaweed species on appetite in healthy participants and reported elevated satiety after 20 min of consuming a meal containing 5 g *U. pinnatifida* and 30 g starch compared to the control meal containing pea protein instead of seaweed. Fullness scores were also higher after 20 and 50 min of meal intake and scores for prospective food consumption score (i.e., how much food the participants thought they could eat) were lower up to 100 min after the meal intake. *Laminaria digitata* was also tested, but apart from a higher fullness score after 20 min compared to the control, no other differences were found. Notably, the seaweed meal contained around 9 times more dietary fiber per serving than the energy-adjusted control meal (1.7 vs. 0.2 g fiber) and the reasons for selecting pea protein to replace seaweed are unknown (38). In another study, consumption of a meal containing 70 g FW of *U. pinnatifida* with rice, soybeans, potatoes, and broccoli vs. the same meal without seaweed did not have any differential effects on appetite scores, such as postprandial feelings of fullness, satisfaction, pleasantness, and hunger (41).

At present, there is insufficient evidence concerning potential effects of seaweed on appetite. The only study favoring the seaweed intervention has only reported immediate effects up to 100 min after meal consumption. Thus, such time may have limited influence on what and how much the individual consumes at subsequent meals.

### 3.9. Effects on thyroid function and urinary iodine

Seaweeds, especially brown species, are sources of iodine - an essential micronutrient for thyroid function as it is a rate-limiting element for the synthesis of the thyroid hormones triiodothyronine (T3) and thyroxine (T4) (77). Concurrently, excessive iodine intake can cause subclinical hypothyroidism characterized by mildly elevated serum thyroid stimulating hormone (TSH), usually above 4.0 mU/L, and normal serum-free T4 levels (78–80). Subclinical hypothyroidism can progress to overt hypothyroidism, particularly in individuals with underlying thyroid disease (e.g., Graves’ disease, Hashimoto’s thyroiditis) and can if left untreated result in, e.g., pericardial effusion, pleural effusion, and subfertility (81). Although we could not find reports directly linking iodine intake with a higher prevalence of hypothyroidism, epidemiological data show that the prevalence of subclinical and overt hypothyroidism is higher in iodine-sufficient areas and in areas traditionally consuming seaweed (79, 81).

Five RCTs evaluated the effects of thyroid function following short-term (40) and semi long-term seaweed consumption (28, 33, 53, 82), and all but one study (28) evaluated urinary iodine excretion. Four out of those five RCTs showed similar findings in terms of minor rises in TSH (below 4.0 mU/L) and constant free T4 levels and detected increases in urinary iodine, if measured.

One study (40) randomized six healthy adults to either consume a sushi meal with a seaweed salad containing 25 g FW of blanched *Fucus vesiculosus* or the same meal without the seaweed, on one single occasion. There was a five-fold increase in urinary iodine excretion after the seaweed meal compared to the control. Furthermore, the iodine load led to a short-term rise in TSH levels (50%), while free T4 levels remained constant until 6.5 days after meal intake (40). Another study (53) observed similar findings after subjecting healthy postmenopausal women to 5 g DW of *A. esculenta*/day (equivalent to 475 μg iodine/day) for 6 weeks compared to placebo capsules containing maltodextrin. Likewise, Takase et al. (33) reported a similar trend in Japanese individuals with hypercholesterolemia after 8 weeks of consuming 2 g DW of *P. palmata*/day (equivalent to 1,424 μg iodine/day) compared to placebo capsules containing porcine gelatin. Identical findings were observed when supplementing healthy participants with 1.2 and 2.4 g DW of kelp/day (equivalent to 660 and 1,320 μg iodine/day, respectively) for 4 weeks compared to baseline, while no differences were found compared to the control group that ingested capsules containing alfalfa (82). Lastly, another study (28) reported similar TSH and T4 levels in healthy individuals after intervention with 0.8 g DW of *Kjellmaniella crassifolia*/day for 8 weeks (iodine load not disclosed) compared to baseline or control (placebo) group.

Overall, intake of iodine present in seaweed at doses ranging from 475 to 1,320 μg of iodine/day did not increase TSH levels above 4.0 mU/L and is deemed unlikely to cause subclinical hypothyroidism in adults when consumed for up to 8 weeks. Importantly, none of the studies reported adverse effects or persistent alterations (8 weeks) in thyroid function, although some doses surpassed the upper tolerable daily intake of iodine recommended by the European Food Safety Agency (600 μg/day) albeit within the acceptable range established from Japanese health authorities (3,000 μg/day) (83, 84). Long-term RCTs are warranted since current literature was limited to 8-week interventions without follow-up.

### 3.10. Miscellaneous

#### 3.10.1. Renal, liver function and blood count

Seaweed intake did not affect renal (26–28, 33) and liver function (26–28, 33), as determined by blood urea nitrogen, creatinine, uric acid, alanine transaminase, aspartate transaminase, gamma-glutamyl transpeptidase, and lactate dehydrogenase. The same was true for blood count (26–28).

#### 3.10.2. Inflammation biomarkers

One study (43) found relatively elevated levels of C-reactive protein (CRP) - an inflammatory marker associated with the risk of cardiovascular disease and mortality (85) - following consumption of *P. palmata* (5 g DW/day) in healthy adults compared to control, though CRP levels were within the normal range. Two other studies investigated inflammatory markers and immune system regulation but did not find any effects of *U. pinnatifida* (4 and 6 g DW/day for 4–8 weeks) (57) or *K. crassifolia* (0.8 g DW/day for 8 weeks) (28).

#### 3.10.3. Neuropsychological functions and physical performance

Fermented *S. japonica* significantly improved the results of neuropsychological and physical tests carried out in elderly participants, but only when compared to the change from baseline to placebo (37). Another trial testing fermented *S. japonica* in middle-aged women did not find improvements in muscle strength parameters albeit a significant increase in total lean muscle mass was noticed compared to the placebo group (29). Similarly, no improvements in subjective physical and mental fatigue were reported after the long-term intake of *K. crassifolia* (28).

#### 3.10.4. Estrogen metabolism

In postmenopausal healthy women, a study (51) reported similar levels of estrogen hormones after intervention with 5 g DW *A. esculenta*/day compared to placebo. However, an exploratory analysis showed an inverse correlation between seaweed dose/kg body weight and estradiol levels.

### 3.11. Results of risk-of-bias assessment

**Figure 3:**
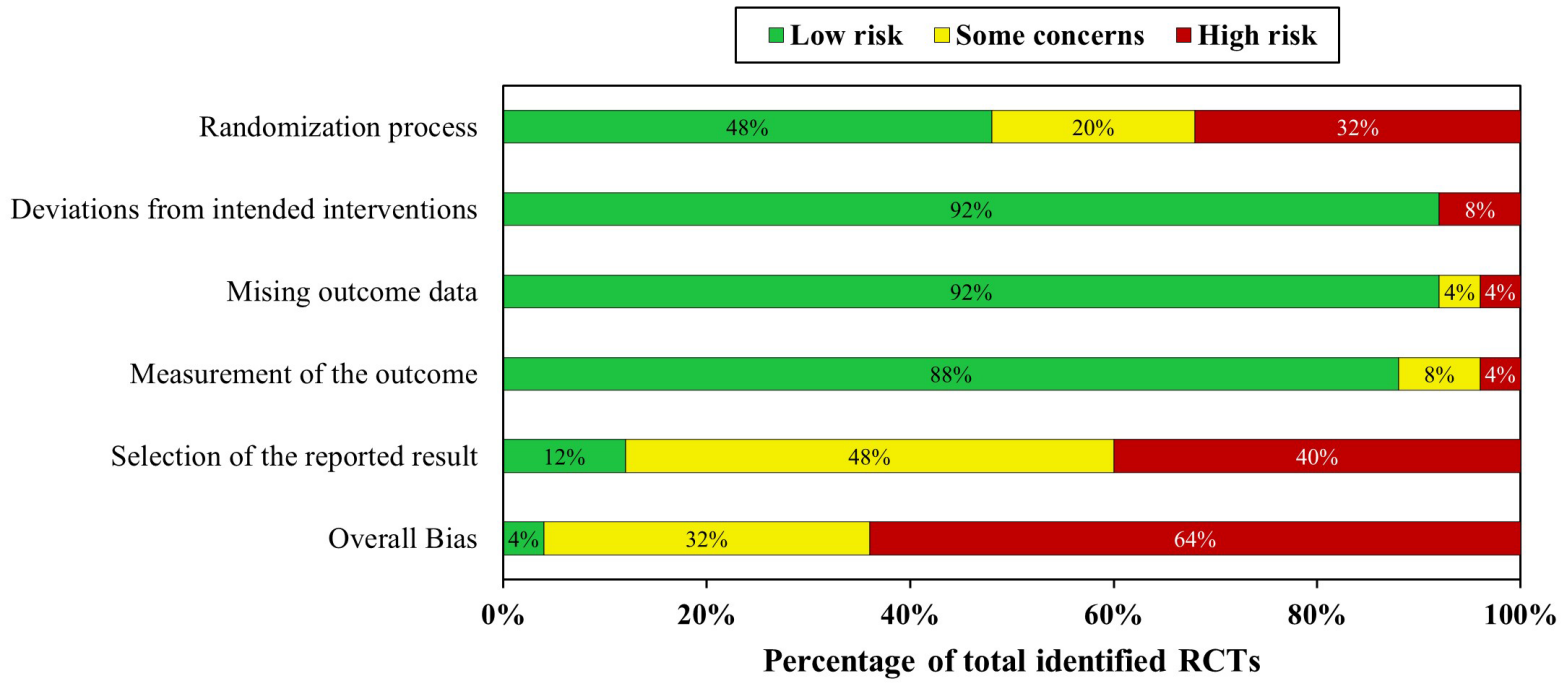
Bias level distribution for each risk-of-bias domain.

Figure 2 shows the risk-of-bias assessment all 25 compiled RCTs. Only one study (4% of the total) was graded with an overall low risk-of-bias (28); 9 studies (36%) were assessed to have some concerns; and the remaining 15 studies (60%) were graded an overall high risk-of-bias. Based on the bias distribution within each domain (Figure 3), bias was judged to mainly arise from the selection of the reported outcome (i.e., domain 5) followed by bias on the randomization process (i.e., domain 1). Concerning the selection of the reported outcome, 12 studies (48%) were graded on some bias concerns and 10 studies (40%) had a high risk-of-bias. The main contributing reasons were related to (i) a lack of pre-specified analysis plans; (ii) multiple eligible outcome measurements within the outcome; and (iii) multiple eligible analyzes of the data. Regarding the randomization process, 5 studies (20%) showed some bias concerns and 8 studies (32%) a high risk-of-bias. Factors contributing to these assessments include (i) no information on allocation or randomization method and (ii) reason to assume the investigator(s) and/or the participants knew of the upcoming intervention. Bias arising from deviations from intended interventions, missing outcome data, or measurement of the outcome (i.e., domains 2 to 4, respectively) were generally deemed low as 22 studies (88%) had a low risk-of-bias.

### 3.12. Strengths and limitations of the compiled studies

The compiled studies exhibited the following strengths: the selected doses aligned with the estimated levels consumed by the Japanese population, enabling easier extrapolation to real-life scenarios; encapsulation was the prevailing delivery method, which ideally should offer blinding of study participants, personnel, and investigators; extensive testing has been conducted on brown seaweed species, creating an opportunity for investigating the effects of red and green seaweeds; at least 22 out of the 25 RCTs had a low risk-of-bias on the domains related to deviations from intended interventions, missing outcome data, and measurement of the outcome.

Despite these strengths, there were some limitations. One issue related to the variation in the pre-treatment and drying methods employed, which hindered comparisons among RCTs that examine the same seaweed species. Additionally, few studies conducted a power calculation or addressed whether the sample size was adequately determined in relation to the desired study outcomes. Lastly, most studies showed bias arising from the selection of the reported outcome and from the randomization process. These biases collectively limit the ability to draw firm conclusions, especially since there was limited literature on selected outcomes.

## 4. Conclusion

This review aimed at investigating the evidence for effects of whole seaweed consumption on humans based on published RCTs. This included multiple outcomes, including those predominantly related to cardiometabolic diseases and risk factors, thyroid function, urinary iodine, and oxidative stress. The aim was further to critically assess the methodology and Cochrane risk-of-bias. We identified 25 RCTs published after the year 2000; most of which investigated the effects of brown seaweed species, commonly encapsulated with varying placebo/control.

In summary, we found limited but favorable evidence for effects of seaweed on blood glucose metabolism, blood pressure, anthropometric measures, and to lesser extent blood lipids. Favorable effects were often observed in populations with type 2 diabetes (for glucose response) and hypertension (for studies on blood pressure). Concerning thyroid function, no adverse effects were observed, and seaweed did not increase TSH levels above the level for subclinical hypothyroidism during short-term consumption (< 8 weeks) for an iodine dose ranging from 475 to 1,320 μg/day. Similarly, seaweed did not affect renal or liver function nor influence blood minerals concentration. Lastly, there was insufficient literature on estrogen metabolism, subjective appetite, neuropsychological functions and physical performance, and inflammatory markers.

Based on the identified knowledge gaps, strengths, and limitations of current literature, future RCTs examining the effects of seaweed intake shall (i) improve the robustness of the study design aiming at low risk-of-bias; (ii) register trial protocols prospectively; (iii) transparently report on methods and provide sufficient information on, e.g., the selection of control diet, blinding protocols and deviations from work plan and; (iv) calculate and report on power calculation. Also, if seaweed is to be integrated into the Western diet it would likely need to be included as a whole food/ingredient rather than a capsule, thus evidence from studies including seaweed in meals or food products may be more applicable to real life. Lastly, we call for RCTs investigating ubiquitous seaweed species cultivated in regions other than Asia, e.g., in Europe or North America, with a focus on green and red species. Such RCTs will complement current literature and can provide further evidence-based support for the increasing use of seaweed in regions with negligible seaweed consumption.

## Author contributions

JT: conceptualized and performed the literature search, identified the studies meeting the inclusion criteria, extracted data from the included studies, and wrote original draft. MP-B assessed the risk of bias of the included studies, assisted in the interpretation of the results, and revised and edited the manuscript. MJ: wrote original draft and revised and edited the manuscript. IU: conceptualized and performed the literature search, identified the studies meeting the inclusion criteria, assisted in the interpretation of the results, revised and edited the manuscript, and acquired funding.

## Funding

The study was supported by Formas and conducted within the projects ‘The role of algae in sustainable food systems’ (Grant no. 2020–03113), CirkAlg (Grant no. 2018–01839), and BlueGreen (Grant no. 2021–02340).

## Conflict of interest

The authors declare that the research was conducted in the absence of any commercial or financial relationships that could be construed as a potential conflict of interest.

## Publisher’s note

All claims expressed in this article are solely those of the authors and do not necessarily represent those of their affiliated organizations, or those of the publisher, the editors and the reviewers. Any product that may be evaluated in this article, or claim that may be made by its manufacturer, is not guaranteed or endorsed by the publisher.
